# Using qualitative Health Research methods to improve patient and public involvement and engagement in research

**DOI:** 10.1186/s40900-018-0129-8

**Published:** 2018-12-13

**Authors:** Danielle E. Rolfe, Vivian R. Ramsden, Davina Banner, Ian D. Graham

**Affiliations:** 1 School of Epidemiology and Public Health, University of Ottawa, 307D- 600 Peter Morand Crescent, Ottawa, ON K1G 5Z3 Canada; 2 Department of Academic Family Medicine, University of Saskatchewan, West Winds Primary Health Centre, 3311 Fairlight Drive, Saskatoon, SK S7M 3Y5 Canada; 3 School of Nursing, University of Northern British Columbia, 3333 University Way, Prince George, BC V2K4C6 Canada

**Keywords:** Qualitative methods, Qualitative health research, Patient-oriented research, Integrated knowledge translation, Patient engagement, Patient partners, Patient and public involvement

## Abstract

**Plain English summary:**

Patient engagement (or patient and public involvement) in health research is becoming a requirement for many health research funders, yet many researchers have little or no experience in engaging patients as partners as opposed to research subjects. Additionally, many patients have no experience providing input on the research design or acting as a decision-making partner on a research team. Several potential risks exist when patient engagement is done poorly, despite best intentions. Some of these risks are that: (1) patients’ involvement is merely tokenism (patients are involved but their suggestions have little influence on how research is conducted); (2) engaged patients do not represent the diversity of people affected by the research; and, (3) research outcomes lack relevance to patients’ lives and experiences.

Qualitative health research (the collection and systematic analysis of non-quantitative data about peoples’ experiences of health or illness and the healthcare system) offers several approaches that can help to mitigate these risks. Several qualitative health research methods, when done well, can help research teams to: (1) accurately incorporate patients’ perspectives and experiences into the design and conduct of research; (2) engage diverse patient perspectives; and, (3) treat patients as equal and ongoing partners on the research team.

This commentary presents several established qualitative health research methods that are relevant to patient engagement in research. The hope is that this paper will inspire readers to seek more information about qualitative health research, and consider how its established methods may help improve the quality and ethical conduct of patient engagement for health research.

**Abstract:**

**Background**

Research funders in several countries have posited a new vision for research that involves patients and the public as co-applicants for the funding, and as collaborative partners in decision-making at various stages and/or throughout the research process. Patient engagement (or patient and public involvement) in health research is presented as a more democratic approach that leads to research that is relevant to the lives of the people affected by its outcomes. What is missing from the recent proliferation of resources and publications detailing the practical aspects of patient engagement is a recognition of how existing research methods can inform patient engagement initiatives. Qualitative health research, for example, has established methods of collecting and analyzing non-quantitative data about individuals’ and communities’ lived experiences with health, illness and/or the healthcare system. Included in the paradigm of qualitative health research is participatory health research, which offers approaches to partnering with individuals and communities to design and conduct research that addresses their needs and priorities.

**Discussion**

The purpose of this commentary is to explore how qualitative health research methods can inform and support meaningful engagement with patients as partners. Specifically, this paper addresses issues of: *rigour* (how can patient engagement in research be done well?); *representation* (are the right patients being engaged?); and, *reflexivity* (is engagement being done in ways that are meaningful, ethical and equitable?). Various qualitative research methods are presented to increase the rigour found within patient engagement. Approaches to engage more diverse patient perspectives are presented to improve representation beyond the common practice of engaging only one or two patients. Reflexivity, or the practice of identifying and articulating how research processes and outcomes are constructed by the respective personal and professional experiences of researchers and patients, is presented to support the development of authentic, sustainable, equitable and meaningful engagement of patients as partners in health research.

**Conclusions**

Researchers will need to engage patients as stakeholders in order to satisfy the overlapping mandate in health policy, care and research for engaging patients as partners in decision-making. This paper presents several suggestions to ground patient engagement approaches in established research designs and methods.

## Background

Patient engagement (or patient and public involvement) in research involves partnering with ‘patients’ (a term more often used in Canada and the US, that is inclusive of individuals, caregivers, and/or members of the public) to facilitate research related to health or healthcare services. Rather than research subjects or participants, patients are engaged as partners in the research process. This partnership is intended to be meaningful and ongoing, from the outset of planning a research project, and/or at various stages throughout the research process. Engagement can include the involvement of patients in defining a research question, identifying appropriate outcomes and methods, collecting and interpreting data, and developing and delivering a knowledge translation strategy [1].

The concept of engaging non-researchers throughout the research process is not new to participatory health researchers, or integrated knowledge translation researchers, as the latter involves ongoing collaboration with clinicians, health planners and policy makers throughout the research process in order to generate new knowledge [2, 3]. Patients, however, are less frequently included as partners on health research teams, or as knowledge users in integrated knowledge translation research teams compared to clinicians, healthcare managers and policy-makers, as these individuals are perceived as having “the authority to invoke change in the practice or policy setting.” (p.2) [2] Recent requirements for patient engagement by health research funders [4–6], ,and mandates by most healthcare planners and organizations to engage patients in healthcare improvement initiatives, suggest that it would be prudent for integrated knowledge translation (and indeed all) health researchers to begin engaging patients as knowledge users in many, if not all, of their research projects.

Training and tools for patient engagement are being developed and implemented in Canada via the Canadian Institutes for Health Research (CIHR) Strategy for Patient Oriented Research (SPOR) initiative, in the US via Patient Centered Outcomes Research Institute (PCORI), and very practical resources are already available from the UK’s more established INVOLVE Advisory Group [5–7]. What is seldom provided by these ‘get started’ guides, however, are rigorous methods and evidence-based approaches to engaging diverse patient perspectives, and ensuring that their experiences, values and advice are appropriately incorporated into the research process.

The purpose of this commentary is to stimulate readers’ further discussion and inquiry into qualitative health research methods as a means of fostering the more meaningfully engagement of patients as partners for research. Specifically, this paper will address issues of: *rigour* (how do we know that the interpretation of patients’ perspectives has been done well and is applicable to other patients?); *representation* (are multiple and diverse patient perspectives being sought?); and, *reflexivity* (is engagement being done ethically and equitably?). This commentary alone is insufficient to guide researchers and patient partners to use the methods presented as part of their patient engagement efforts. However, with increased understanding of these approaches and perhaps guidance from experienced qualitative health researchers, integrated knowledge translation and health researchers alike may be better prepared to engage patients in a meaningful way in research that has the potential to improve health and healthcare experiences and outcomes.

### What can be learned from methods utilized in qualitative health research?

There is wide variation in researchers’ and healthcare providers’ openness to engaging patients [8]. Often, the patients that are engaged are a select group of individuals known to the research team, sometimes do not reflect the target population of the research, are involved at a consultative rather than a partnership level, and are more likely to be involved in the planning rather than the dissemination of research [9–11]. As a result, patient engagement can be seen as tokenistic and the antithesis of the intention of most patient engagement initiatives, which is to have patients’ diverse experiences and perspectives help to shape what and how research is done. The principles, values, and practices of qualitative health research (e.g., relativism, social equity, inductive reasoning) have rich epistemological traditions that align with the conceptual and practical spirit of patient engagement. It is beyond the scope of this commentary, however, to describe in detail the qualitative research paradigm, and readers are encouraged to gain greater knowledge of this topic via relevant courses and texts. Nevertheless, several qualitative research considerations and methods can be applied to the practice of patient engagement, and the following sections describe three of these: rigour, representation and reflexivity.

### Rigour: Interpreting and incorporating patients’ experiences into the design and conduct of research

When patient engagement strategies go beyond the inclusion of a few patient partners on the research team, for example, by using focus groups, interviews, community forums, or other methods of seeking input from a broad range of patient perspectives, the diversity of patients’ experiences or perspectives may be a challenge to quickly draw conclusions from in order to make decisions about the study design. To make these decisions, members of the research team (which should include patient partners) may discuss what they heard about patients’ perspectives and suggestions, and then unsystematically incorporate these suggestions, or they may take a vote, try to achieve consensus, implement a Delphi technique [12], or use another approach designed specifically for patient engagement like the James Lind Alliance technique for priority setting [13]. Although the information gathered from patients is not data (and indeed would require ethical review to be used as such), a number of qualitative research practices designed to increase rigour can be employed to help ensure that the interpretation and incorporation of patients’ experiences and perspectives has been done systematically and could be reproduced [14]. These practices include *member checking*, *dense description*, and *constant comparative analysis*. To borrow key descriptors of rigour from qualitative research, these techniques improve “credibility” (i.e., accurate representations of patients’ experiences and preferences that are likely to be understood or recognized by other patients in similar situations – known in quantitative research as internal validity), and “transferability” (or the ability to apply what was found among a group of engaged patients to other patients in similar contexts – known in quantitative research as external validity) [15].

### Member checking

Member checking in qualitative research involves “taking ideas back to the research participants for their confirmation” (p. 111) [16]. The objective of member checking is to ensure that a researcher’s interpretation of the data (whether a single interview with a participant, or after analyzing several interviews with participants) accurately reflects the participants’ intended meaning (in the case of a member check with a single participant about their interview), or their lived experience (in the case of sharing an overall finding about several individuals with one or more participants) [16]. For research involving patient engagement, member checking can be utilized to follow-up with patients who may have been engaged at one or only a few time points, or on an on-going basis with patient partners. A summary of what was understood and what decisions were made based on patients’ recommendations could be used to initiate this discussion and followed up with questions such as, “have I understood correctly what you intended to communicate to me?” or “do you see yourself or your experience(s) reflected in these findings or suggestions for the design of the study?”

### Dense description

As with quantitative research, detailed information about qualitative research methods and study participants is needed to enable other researchers to understand the context and focus of the research and to establish how these findings relate more broadly. This helps researchers to not only potentially repeat the study, but to extend its findings to similar participants in similar contexts. Dense description provides details of the social, demographic and health profile of participants (e.g., gender, education, health conditions, etc.), as well as the setting and context of their experiences (i.e., where they live, what access to healthcare they have). In this way, dense description improves the transferability of study findings to similar individuals in similar situations [15]. To date, most studies involving patient engagement provide limited details about their engagement processes and who was engaged [17]. This omission may be done intentionally (e.g., to protect the privacy of engaged patients, particularly those with stigmatizing health conditions), or as a practical constraint such as publication word limits. Nonetheless, reporting of patient engagement using some aspects of dense description of participants (as appropriate), the ways that they were engaged, and recommendations that emanated from engaged patients can also contribute to greater transferability and understanding of how patient engagement influenced the design of a research study.

### Constant comparative analysis

Constant comparative analysis is a method commonly used in grounded theory qualitative research [18]. Put simply, the understanding of a phenomenon or experience that a researcher acquires through engaging with participants is constantly redeveloped and refined based on subsequent participant interactions. This process of adapting to new information in order to make it more relevant is similar to processes used in rapid cycle evaluation during implementation research [19]. This method can be usefully adapted and applied to research involving ongoing collaboration and partnership with several engaged patient partners, and/or engagement strategies that seek the perspectives of many patients at various points in the research process. For example, if, in addition to having ongoing patient partners, a larger group of patients provides input and advice (e.g., a steering or advisory committee) at different stages in the research process, their input may result in multiple course corrections during the design and conduct of the research processes to incorporate their suggestions. These suggestions may result in refinement of earlier decisions made about study design or conduct, and as such, the research process becomes more iterative rather than linear. In this way, engaged patients and patient partners are able to provide their input and experience to improve each step of the research process from formulating an appropriate research question or objective, determining best approaches to conducting the research and sharing it with those most affected by the outcomes.

### Representation: Gathering diverse perspectives to design relevant and appropriate research studies

The intention of engaging patients is to have their lived experience of health care or a health condition contribute to the optimization of a research project design [20]. Development of a meaningful and sustainable relationship with patient partners requires considerable time, a demonstrated commitment to partnership by both the patient partners and the researcher(s), resources to facilitate patient partners’ engagement, and often, an individual designated to support the development of this relationship [17, 21]. This may lead some research teams to sustain this relationship with only one or two patients who are often previously known to the research team [17]. The limitation of this approach is that the experiences of these one or two individuals may not adequately reflect the diverse perspectives of patients that may be affected by the research or its outcomes. The notion of gaining ‘*the* patient perspective’ from a single or only a few individuals has already been problematized [22, 23]. To be sure, the engagement of a single patient is better than none at all, but the engagement of a broader and diverse population of patients should be considered to better inform the research design, and to help prevent further perpetuation of health disparities. Key issues to be considered include (1) how engagement can be made accessible to patients from diverse backgrounds, and (2) which engagement strategies (e.g., ranging from a community information forum to full partnership on the research team) are most appropriate to reach the target population [24].

### Making engagement accessible

Expecting patient partner(s) to attend regular research team meetings held during working hours in a boardroom setting in a hospital, research institute or university limits the participation of many individuals. To support the participation and diversity of engaged patients, effort should be made to increase the accessibility and emotional safety of engagement initiatives [25]. A budget must be allocated for patient partners’ transportation, childcare or caregiving support, remuneration for time or time taken off work and, at the very least, covering expenses related to their engagement. Another consideration that is often made by qualitative health researchers is whether brief counselling support can be provided to patients should the sharing of their experiences result in emotional distress. There are some resources that can help with planning for costs [26], including an online cost calculator [27].

### Engagement strategies

Patient partners can be coached to consider the needs and experiences of people unlike them, but there are other methods of engagement that can help to gain a more fulsome perspective of what is likely a diverse patient population that is the focus of the research study. In qualitative health research, this is known as purposeful or purposive sampling: finding people who can provide information-rich descriptions of the phenomenon under study [28]. Engagement may require different approaches (e.g., deliberative group processes, community forums, focus groups, and patient partners on the research team), at different times in the research process to reach different individuals or populations (e.g., marginalized patients, or patients or caregivers experiencing illnesses that inhibit their ability to maintain an ongoing relationship with the research team). Engagement strategies of different forms at different times may be required. For example, ongoing engagement may occur with patient partners who are members of the research team (e.g., co-applicants on a research grant), and intermittent engagement may be sought from other patients through other methods that may be more time-limited or accessible to a diverse population of patients (e.g., a one-time focus group, community forum, or ongoing online discussion) to address issues that may arise during various stages of the research or dissemination processes. The result of this approach is that patients are not only consulted or involved (one-time or low commitment methods), but are also members of the research team and have the ability to help make decisions about the research being undertaken.

Engagement can generate a wealth of information from very diverse perspectives. Each iteration of engagement may yield new information. Knowing when enough information has been gathered to make decisions with the research team (that includes patient partners) about how the research may be designed or conducted can be challenging. One approach from qualitative research that can be adapted for patient engagement initiatives is theoretical saturation [29], or “the point in analysis when…further data gathering and analysis add little new to the conceptualization, though variations can always be discovered.” (p. 263) [18]. That is, a one-time engagement strategy (e.g., a discussion with a single patient partner) may be insufficient to acquire the diverse perspectives of the individuals that will be affected by the research or its outcomes. Additional strategies (e.g., focus groups or interviews with several individuals) may be initiated until many patients identify similar issues or recommendations.

Engagement approaches should also consider: how patients are initially engaged (e.g., through known or new networks, posted notices, telephone or in-person recruitment) and whether involvement has been offered widely enough to garner multiple perspectives; how patients’ experiences are shared (e.g., community forums, formal meetings, individual or group discussions) and whether facilitation enables broad participation; and finally, how patients’ participation and experiences are incorporated into the research planning and design, with patients having equal decision-making capacity to other research team members. Several publications and tools are available that can help guide researchers who are new to processes of engaging patients in research [24, 30–34], but unfortunately few address how to evaluate the effectiveness of engagement [35].

### Reflexivity: Ensuring meaningful and authentic engagement

In qualitative research, reflexivity is an ongoing process of “the researcher’s scrutiny of his or her research experience, decisions, and interpretations in ways that bring the researcher into the process and allow the reader to assess how and to what extent the researcher’s interests, positions, and assumptions influenced inquiry. A reflexive stance informs how the researcher conducts his or her research, relates to the research participants, and represents them in written reports,” (p.188–189) [16]. The concept of reflexivity can be applied to research involving patient engagement by continually and explicitly considering how decisions about the research study were made. All members of the research team must consider (and perhaps discuss): (1) how patient partners are invited to participate in research planning and decision-making; (2) how their input is received relative to other team members (i.e., do their suggestions garner the same respect as researchers’ or providers’?); and, (3) whether engaged patients or patient partners feel sufficiently safe, able and respected to share their experiences, preferences and recommendations with the research team.

Ideally, reflexivity becomes a practice within the research team and may be operationalized through regular check-ins with patients and researchers about their comfort in sharing their views, and whether they feel that their views have been considered and taken onboard. Power dynamics should also be considered during patient engagement initiatives. For example, reflecting on how community forums, focus groups or interviews are to be facilitated, including a consideration of who is at the table/who is not, who speaks/who does not, whose suggestions are implemented/whose are not? Reflexivity can be practiced through informal discussions, or using methods that may allow more candid responses by engaged patients (e.g., anonymous online survey or feedback forms). At the very least, if these practices were not conducted throughout the research process, the research team (including patient partners) should endeavor to reflect upon team dynamics and consider how these may have contributed to the research design or outcomes. For example, were physicians and researchers seen as experts and patients felt less welcome or able to share their personal experiences? Were patients only engaged by telephone rather than in-person and did this influence their ability to easily engage in decision-making? Reflexive practices may be usefully supplemented by formal evaluation of the process of patient engagement from the perspective of patients and other research team members [36, 37], and some tools are available to do this [35].

### A note about language

One way to address the team dynamic between researchers, professional knowledge users (such as clinicians or health policy planners) and patients is to consider the language used to engage with patients in the planning of patient engagement strategies. That is, the term ‘patient engagement’ is a construction of an individual’s identity that exists only within the healthcare setting, and in the context of a patient-provider dynamic. This term does not consider how people make decisions about their health and healthcare within a broader context of their family, community, and culture [22, 38]. This may be why research communities in some countries (e.g., the United Kingdom) use the term ‘patient and public involvement’. Additionally, research that involves communities defined by geography, shared experiences, cultural or ethnic identity, as is the case with participatory health research, may refer to ‘community engagement.’ Regardless of the term used, partnerships with patients, the public, or with communities need to be conceived instead as person-to-person interactions between researchers and individuals who are most affected by the research. Discussions with engaged patients should be conducted early on to determine how to best describe their role on the team or during engagement initiatives (e.g., as patient partners, community members, or people with lived experience).

### Tokenism

Tokenism is the “difference between…the empty ritual of participation and having the real power needed to affect the outcome,” (p.2) [39]. Ongoing reflection on the power dynamic between researchers and engaged patients, a central tenet of critical qualitative health research [40, 41], can increase the likelihood that engagement involves equitable processes and will result in meaningful engagement experiences by patients rather than tokenism [36, 42]. Patient engagement initiatives should strive for “partnership” amongst all team members, and not just reflect a patient-clinician or researcher-subject dynamic [43]. To develop meaningful, authentic and sustainable relationships with engaged patients, methods used for participatory, action or community-based research (approaches that fall under the paradigm of qualitative inquiry) provide detailed experiential guidance [44]. For example, a realist review of community-based participatory research projects reported that gaining and maintaining trust with patient or community partners, although time-intensive, is foundational to equitable and sustainable partnerships that benefit communities and individuals [45, 46]. Additionally, Chapter Nine of the Canadian Tri-Council Policy Statement on Research involving Humans, which has to date been applied to research involving First Nations, Inuit and, Métis Peoples in Canada [47], provides useful information and direction that can be applied to working with patient partners on research [48].

Authentic patient engagement should include their involvement at all stages of the research process [49, 50], but this is often not the case [10]. .Since patient partners are not research subjects or participants, their engagement does not (usually) require ethics approval, and they can be engaged as partners as early as during the submission of grant applications [49]. This early engagement helps to incorporate patients’ perspectives into the proposed research before the project is wedded to particular objectives, outcomes and methods, and can also serve to allocate needed resources to support patient engagement (including remuneration for patient partners’ time). Training in research for patient partners can also support their meaningful engagement by increasing their ability to fully engage in decision-making with other members of the research team [51, 52]. Patient partners may also thrive in co-leading the dissemination of findings to healthcare providers, researchers, patients or communities most affected by the research [53].

## Conclusion

Patient engagement has gained increasing popularity, but many research organizations are still at the early stages of developing approaches and methods, many of which are based on experience rather than evidence. As health researchers and members of the public will increasingly need to partner for research to satisfy the overlapping mandate of patient engagement in health policy, healthcare and research, the qualitative research methods highlighted in this commentary provide some suggestions to foster rigorous, meaningful and sustained engagement initiatives while addressing broader issues of power and representation. By incorporating evidence-based methods of gathering and learning from multiple and diverse patient perspectives, we will hopefully conduct better patient engaged research, live out the democratic ideals of patient engagement, and ultimately contribute to research that is more relevant to the lives of patients; as well as, contribute to the improved delivery of healthcare services. In addition to the references provided in this paper, readers are encouraged to learn more about the meaningful engagement of patients in research from several key texts [54–56].

## Abbreviations

CIHR: Canadian Institutes for Health Research
PCORI: Patient Centered Outcomes Research Institute
SPOR: Strategy for Patient Oriented Research
